# Transformation of *Penicillium rubens* 212 and Expression of GFP and DsRED Coding Genes for Visualization of Plant-Biocontrol Agent Interaction

**DOI:** 10.3389/fmicb.2018.01653

**Published:** 2018-07-23

**Authors:** Maria Villarino, Eduardo A. Espeso, Paloma Melgarejo, Inmaculada Larena

**Affiliations:** ^1^Departamento de Protección Vegetal, Subdirección General de Investigación y Tecnología (SGIT), Instituto Nacional de Investigación y Tecnología Agraria y Alimentaria (INIA), Madrid, Spain; ^2^Departamento de Biología Celular y Molecular, Centro de Investigaciones Biológicas (CIB), Consejo Superior de Investigaciones Científicas, Madrid, Spain

**Keywords:** *DsRed*, *GFP*, fluorescent reporter, biocontrol, root colonization, fungal transformation, autonomous replication

## Abstract

Strain 212 of *Penicillium rubens* (PO212) is an effective fungal biological control agent against a broad spectrum of diseases of horticultural plants. A pyrimidine auxotrophic isolate of PO212, PO212*_*18.2, carrying an inactive *pyrG* gene, has been used as host for transformation by positive selection of vectors containing the gene complementing the *pyrG1* mutation. Both integrative and autonomously replicating plasmids transformed PO212*_*18.2 with high efficiency. Novel PO212-derived strains expressed green (*sGFP)* and red (Ds-Red Express) fluorescent reporter proteins, driven by the *A. nidulans gpdA* promoter. Fluorescence microscopy revealed constitutive expression of the sGFP and Ds-Red Express proteins, homogenously distributed across fungal cells. Transformation with either type of plasmid, did not affect the growth and morphological culture characteristics, and the biocontrol efficacy of either transformed strains compared to the wild-type, PO212. Fluorescent transformants pointed the capacity of PO212 to colonize tomato roots without invading plant root tissues. This work demonstrates susceptibility of the biocontrol agent PO212 to be transformed, showing that the use of GFP and DsRed as markers for PO212 is a useful, fast, reliable and effective approach for studying plant–fungus interactions and tomato root colonization.

## Introduction

The isolate 212 of *Penicillium rubens* (PO212, ATCC 201888), formerly *Penicillium oxalicum* (Villarino et al., 2016), has proven to be an effective biological control agent (BCA) against a broad spectrum of diseases of horticultural plants (Larena et al., 2003; De Cal et al., 2008, 2009; Martinez-Beringola et al., 2013) including tomato *Fusarium* wilt caused by *Fusarium oxysporum* f. sp. *lycopersici* (FOL) that colonizes and penetrates tomato roots. The disease control is based on a mechanism of induced resistance in tomato plants (De Cal et al., 2000). Furthermore, the application of a conidial suspension of PO212 to the tomato seedlings induces growth promotion in plants (Melgarejo et al., 2005, Patent 200502822). As far as we know, currently only the use of dry mycelium of *Penicillium chrysogenum* as BCA against *Fusarium* and *Verticillium* wilt has been described (Dong and Cohen, 2001, 2002), and there are few cases of *Penicillium* sp. as BCA *in vivo* such as two isolates of *P. citrinum* described as potential fungal agents of *Claviceps africana* in sorghum panicles in glasshouse (Bhuiyan et al., 2003). The work by Peng and Sutton reported to 79–90% incidence reduction of *Botrytis cinerea* on strawberry flowers by conidial suspensions of *Penicillium* spp., *Trichoderma viride* and *Epicoccum nigrum* (Peng and Sutton, 1991), and *P. funiculosum* suppressed *Phytophthora* root rots of azalea (*Rhododendron* spp.) and sweet orange (*Citrus sinensis*) in greenhouse (Fang and Tsao, 1995).

To exploit broadly the biotechnological potential of PO212 as BCA, a deep characterization of this strain is required. For example, in order to control and optimize the application of PO212, it is essential to increase knowledge of the PO212-plant-pathogen interaction. The labeling of BCAs with fluorescent proteins and advances in microscopy for *in situ* monitoring provide great opportunities to study the complex mechanisms of interaction between antagonistic fungi, pathogens and plants (Lu et al., 2004; Bolwerk et al., 2005; Grunewaldt et al., 2007). More knowledge on the *in vivo* interactions between the BCA, pathogen and the plant would help to improve the efficiency of diseases control by the BCA. Prior to the study of this tripartite interaction, it is a prerequisite to study the competent root and rhizosphere colonization by the BCA. This will allow the enhancement of techniques for utilization and application of BCA in a more efficient way.

Various transformation systems for filamentous fungi have been developed with a variety of selectable markers, including antibiotic-resistance and auxotrophic markers (Tilburn et al., 1983; van Hartingsveldt et al., 1987; Woloshuk et al., 1989; Skory et al., 1990; d′Enfert, 1996) and also the novel CRISPR/Cas9-based transformation procedure is being used with success in these organisms (Deng et al., 2017; Zheng et al., 2017). Genetic transformation of biocontrol fungal strains of *Trichoderma virens* (formerly *Gliocladium virens*) (Ossanna and Mischke, 1990), *Trichoderma harzianum* (Thrane et al., 1995) and *Clonostachys rosea* (Lübeck et al., 2002) has become possible, allowing the manipulation of biocontrol fungi at the molecular level. Genetic engineering of *T. harzianum* with reporter and marker genes has provided useful tools for detection and monitoring of introduced BCA in natural environments against a range of soilborne plant pathogens (see for example Green and Jensen, 1995; Thrane et al., 1995; Lo et al., 1998; Bae and Knudsen, 2000).

Transformation procedures for *Penicillium* spp. have been developed, and in this work we approach transformation of a BCA belonging to this genera. In *P. chrysogenum* strains transformation and selection strategies included antibiotic-resistance genes as selection markers either alone (oligomycin, phleomycin, benomyl) or in combination with markers allowing high copy integrations, (i.e., acetamidase coding gene *amdS*) (Beri and Turner, 1987; Bull et al., 1988; Kolar et al., 1988; Picknett and Saunders, 1989). Other selection strategies were based on complementation of auxotrophies with wild-type copies of mutated genes from diverse fungal origins using auxotrophic markers such as *pyrG* (encoding orotidine-5'-phosphate decarboxylase) (Díez et al., 1987; Cantoral et al., 1993; Fierro et al., 1996; Bañuelos et al., 2003), and *niaD* (encoding nitrate reductase) (Whitehead et al., 1989; Gouka et al., 1991). These techniques have been adapted to *Penicillium* (*digitatum* and *expansum*) pathogen strains, allowing the manipulation of these fungi at the genetic level (Buron-Moles et al., 2012).

With respect to the BCA PO212, in a previous work, we obtained mutant PO212 strains resistant to 5-fluoroorotic (5-FOA) acid. Mutations causing tolerance to 5-FOA were isolated at either of two genes of the pyrimidine biosynthetic pathway, *pyrF* and *pyrG* (Villarino et al., 2016). Strain PO212_18.2 carries a mutation in *pyrG* gene (*pyrG1*) causing an early truncation of orotidine-5'-phosphate decarboxylase, consequently becoming a pyrimidine auxotroph. Here we present our advances in developing modified versions of BCA PO212 to enable in depth studies on the mode of action, the ecology, and fitness of this strain under controlled conditions in a laboratory. These and future transformation assays would serve to better understand the behavior of this BCA in host. Thus, we have focused on elaborating a transformation procedure for PO212. Since the genetic basis of biocontrol remains unknown, we used autoreplicative plasmid to preserve the integrity of genome and compared to using a standard integrative plasmid. Both types of plasmids carry homologs of Pr-*pyrG* gene from either *Aspergillus fumigatus* or *Neurospora crassa* species as selectable markers for maintenance in a PO212_18.2 strain. Empty plasmids and *gpdA*-driven fluorescent protein coding genes, GFP and DsRed, have been introduced in PO212 and their effects on biocontrol efficacy, the ability of heterologous expression of proteins and PO212-plant interaction were analyzed.

## Materials and methods

### Strains, media and culture conditions

*Penicillium rubens* PO212 (ATCC 201888) isolated from Spanish soil, has been used in this study and we referred in the text as the wild-type (wt) PO212 strain. wtPO212 was used as a reference and source of DNA for the genomic sequencing of genes under study. PO212_18.2 strain carries the *pyrG1* mutation and was isolated in a previous screening for pyrimidine auxotrophs (Villarino et al., 2016). PO212_18.2 used as recipient for transformation.

Wild-type and recombinant PO212 strains were stored at −80°C in 20% glycerol (long-term storage) and at 4°C on potato dextrose agar (PDA; Difco, Detroit, MI, USA) slants in the dark (short-term storage). All strains generated from wtPO212 were propagated at 22–25°C on PDA, *Aspergillus nidulans* complete medium (Cove, 1966), or *A. nidulans* minimal medium (AMM) with 5 mM ammonium tartrate as nitrogen source and D-glucose 1% (w/v) as carbon source. Uridine (1.22 mg/ml) or uracil (0.56 mg/ml) was added when appropriated to complement the phenotype of *pyrG1* mutation. Cultures used for transformation or DNA preparation were grown in liquid AMM. Transformed strains were maintained on PDA and were also incubated at 20–25°C in the dark for 7 days to obtain heavily sporulating cultures.

Two isolates (0C and 1A) of *Fusarium oxysporum* f. sp*. lycopersici* (Sacc) Snyder and Hansen (FOL) provided by Dr. Cristina Moyano (Laboratory for Assessment of Variety, Seed and Nursery Plants, DTEVP, INIA) used as pathogenic strains and were stored at 4°C in tubes that contained sterile sand, and were grown on Czapek-Dox Agar (CDA) (Difco; Detroit, MI, USA) in the dark at 25°C to mycelial production. Microconidia of FOL (10^5^ microconidias/ml) were produced in flasks containing sterile Czapek-Dox broth (CDB) (Difco; Detroit, MI, USA) each inoculated with mycelial plugs of FOL taken from 7-day-old cultures on CDA. The flasks were incubated at 25°C for 5 days at 150 rpm on a rotary shaker (Model 3527; Lab-Line Instruments, Inc.). The culture was filtered through glass wool and the filtrate used as inoculum (De Cal et al., 1995).

*Escherichia coli* strain DH5α and DH1 were used as host for plasmid propagation and grown in Luria Bertani (LB) liquid media at 37°C. Kanamycin (50 μg/ml) or ampicillin (100 μg/ml) was added to the growth medium when required.

### Plasmids

Two types of plasmids were used for PO212 transformation (Figure S1). In addition, plasmids lacking the gene coding for the fluorescent protein and/or the promoter were included in the transformation process to ensure that the presence of the vector backbone does not interfere in PO212 behavior.

#### Autonomously replicating plasmids

pRG3-AMA1-NotI plasmid (*p1393* in our collection) is an autonomous replicating plasmid carrying *Neurospora crassa pyr4* gene as selectable marker and *AMA1* region of *A. nidulans* (Osherov et al., 2000). This promoter/DsRed-less plasmid was used as control for the transformation process and in biocontrol efficacy assays.

pRG3-gpdA-DsRed plasmid (*p1394*) is an autonomous replicating plasmid, derived from pRG3-AMA1-NotI plasmid, carrying a cassette composed of the constitutive *A. nidulans* glyceraldehyde-3-phosphate dehydrogenase promoter (*gpdA*) and the gene coding for DsRed fluorescent protein (Mikkelsen et al., 2003) and *N. crassa pyr4* gene as selectable marker (Suelmann et al., 1997; Ukil et al., 2009).

#### Integrative plasmids

pFNO3 (*p1439*) is a pCRII-TOPO derived plasmid that contains a cassette encoding for a promoter and ATG-less chimera consisting in a repetition of five Gly-Ala residues (5GA) fused in frame to the sGFP, and followed by the *A. fumigatus pyrG* gene used here as the selectable marker. pFNO3 plasmid has been largely used in *A. nidulans* for the generation of chimeras carrying at the C-terminus the GA5-sGFP tag (Yang et al., 2004; Szewczyk et al., 2006). Here, in transformation of PO212, the integrative promoter-less pFNO3 plasmid is used as a control of integration of a non-expressed copy of GFP in PO212 genome.

*pgpdA5GAGFP* plasmid was constructed using *p1439* as a recipient vector. The gpdA^*mini*^ promoter, a 356 bp fragment, and an ATG codon were inserted before the coding sequence for the GA5-sGFP chimera. The constitutive promoter *gpdA* was obtained from plasmid *p1660* (Pantazopoulou and Penalva, 2009).

### Isolation and manipulation of genomic DNA

The isolation and manipulation of DNA samples was performed as described in Etxebeste et al. (2009). DNA samples were stored at −20°C until required.

### Transformation and positive selection procedures for PO212 transformants

The auxotroph mutant *pyrG1*, PO212_18.2 derived from wtPO212 (Villarino et al., 2016), was transformed as described for *A. nidulans* by Tilburn et al. (1983), with the autonomously replicating plasmids *p1393*, and *p1394*, and also with integrative plasmids *p1439* and *pgpdA5GAGFP*. Essentially, protoplasts of PO212_18.2 were produced by using a mixture of cell wall degrading enzymes, Vinoflow (Novozymes) or Glucanex 100G (Novozymes), purified by a two steps gradient hyperosmotic solution and transformed with circular plasmids using poly ethylene glycol (PEG). Protoplasts were regenerated on AMM containing 1 M sucrose as osmotic stabilizer and supplemented with 1% D-glucose and 5 mM ammonium tartrate as carbon and nitrogen sources, respectively. Non selective regeneration of *pyrG1* protoplasts was done on regeneration medium supplemented with uracil and uridine. Positive transformants were isolated on regeneration medium lacking uracil and uridine.

### Molecular analysis of the transformants

Production of fluorescent proteins by transformants of PO212 was confirmed by Western blot analysis using anti-fluorescent protein antibodies. The protocol used for total protein extraction was the alkaline lysis extraction procedure used for *A. nidulans* described in Hervás-Aguilar and Penalva (2010). Briefly, 6 mg of pulverized mycelium was resuspended in 1 ml buffer (0.2 M NaOH, 0.2% β-mercaptoethanol) and usually 5 μl of each sample were loaded on polyacrylamide gels for protein-content evaluation and estimation of concentration (Hernández-Ortiz and Espeso, 2013). Proteins were then resolved in either 10% SDS-polyacrylamide gels and subsequently transferred to nitrocellulose filters using TransBlot® Turbo™ Transfer System (Bio-Rad). Ds-Red protein was detected using rabbit anti-mRFP (1/4,000; US Biological) and GFP using mouse anti-GFP (1/5,000; polyclonal 7.1 and 13.1; Sigma-Aldrich) as primary antibodies. Actin, detected with mouse anti-γ actin antibody (1/50,000; C4 clone, ICN Biomedicals), was used as loading control. Peroxidase-conjugated goat anti-mouse IgG immunoglobulin (Jackson ImmunoResearch Laboratories) at Correct to 1/4,000 and donkey anti-rabbit IgG immunoglobulin (GE Healthcare) at 1/10,000 were used as secondary antibodies. Peroxidase activity was detected with Amersham Biosciences ECL kit following manufacturer indications.

To characterize transformants carrying integrative plasmids we extracted genomic DNA from mycelia of selected strains grown in liquid AMM. We expected random integration at the genome and the presence of a single copy of the construct was verified by PCR using PrimerStarHS polymerase from Takara (Clontech/Takara Bio Europe) following manufacturer instructions for amplification or short and long DNA fragments. For those transformants carrying the *gpdA*^*mini*^-*gfp/pyrG*^*Af*^ construct we used primers gpdAprosec (5′-TCAGTTCGAGCTTTCCC-3′), annealing at *gpdA* promoter, and MP222 (5′-ATATATCCCGGGTTATTTGTATAGTTCATCCATGC-3′), annealing at the 3′ end of coding sequence for GFP, to verify the amplification of a 825 bp fragment. To analyze those transformants carrying the promoter-less *gfp/pyrG*^*Af*^ construct we used primers Dbp5gsp6^*^ (5′-CGCGCTCAGGCTGGTTTCCGAGGAGCTGGTGCAGGCGCTGGAGCC, annealing at the 5′ end of 5GAGFP coding region, and MP222, and confirmed the amplification of a 760 bp fragment. In case of presence of tandem copies of *pgpdA5GAGFP* and *p1439* plasmids we expected fragments of 7.6 and 7.2 kbp, respectively. Selected strains evidenced the presence of a single integration event of the construct.

### Ecophysiological characterization of transformed strains

Four transformant strains were selected for this work. Two strains called PO212_ar1 and PO212_arRED3, carrying autonomously replicating plasmids pRG3-AMA1-NotI and pRG3-gpdA-DsRed, respectively. And two strains called PO212_in5 and PO212_inGFP9, carrying integrative plasmids pFNO3 and *pgpdA5GAGFP*, respectively. The cultural characteristics of wtPO212 and the transformed strains were determined to identify possible phenotypic differences among them. For this reason growth on PDA, germination and length of germ tubes on potato dextrose broth (PDB; Difco, Detroit, MI, USA) at different pH and temperatures were evaluated (Pascual et al., 1997).

For growth studies *in vitro* a 10 μl droplet of conidia suspension of each strain (10^6^ conidia/ml) on a Viscous Agar (0.2%) and Tween 80 (20%) solution was inoculated in the center of Petri dishes with PDA adjusted at four different pH (4, 5.5, 7, and 8) and incubated at 25°C. Control dishes correspond to PDA adjusted to pH 5.5. Under the same conditions, PDA dishes were inoculated and subsequently incubated at four different temperatures (4, 15, 25, and 35°C). Control dishes corresponded to the incubation at 25°C.

Two perpendicular measurements of the colony diameter, expressed as mm/day were taken at 3, 4, 5, 7, 10, and 14 days after the inoculation. 10 repetitions were performed for each pH, temperature and strain. Each assay was done twice.

To determine conidia germination and length of germ tubes studies *in vitro*, asexual spores of each strain were tested in PDB or CDB by the bioassay described in Larena et al. (2002). For the assay with different pH, CDB was adjusted to four different pH (4, 5.5, 7, and 8) and incubated for 16 h at 20–25°C in darkness. For the assay with different temperatures, each CDB dish was incubated at 4, 15, 25, and 35°C without adjusting pH. Then the percentage of germination of 50 conidia and the length of germ tubes of 25 conidia were counted in each replicate. Three replicate (drops) were made for each treatment (3 repetitions) and the complete experiment was repeated twice. A spore was considered germinated when a germ tube was longer than the length of the spore (Larena et al., 2002).

### Biocontrol efficacy experiments on tomato plants

Assays were performed on tomato plants in growth chambers as described in Larena and Melgarejo (2009) in order to verify if transformation did not affect the biocontrol efficacy compared to wtPO212. One transformant selected from each transformation with plasmids described above were used. Two assays were carried out: (i) the first assay (assay 1) grouped the strains wtPO212, PO212_ar1, PO212_arRED3 and PO212_in5, and (ii) the second assay (assay 2) grouped the strains wtPO212 and PO212_inGFP9. Tomato seeds from cultivar San Pedro was used in the all efficacy experiments and prepared as described by Larena et al. (2003). Tomato seeds were sowed in trays (27 × 42 × 7 cm) with an autoclaved mixture of vermiculite and peat (1:1, v/v). The trays were maintained in a growth chamber at 22–28°C with fluorescent light (100 μE/m^2^ s, 16 h photoperiod) and 80–100% relative humidity for 3 weeks.

Tomato seedlings were treated 7 days before transplanted with an aqueous conidial suspension of each PO212 strain (6 × 10^6^ conidia per gram of substrate). Conidia for the wtPO212 treatment were produced in fermentation bags with a mixture of vermiculite:peat:lentil meal (1:1:0.5, wt/wt/wt, 40% moisture) that were incubated at 20–25°C in the darkness for 5 days as previously described by Larena et al. (2007). Conidia from transformed strains (PO212_ar1, PO212_arRED3, PO212_in5, and PO212_inGFP9) were prepared from suspension of cultures grown on PDA at 20–25°C in the darkness for 7 days. At the time of treatment, the viability of the conidia of each of the treatments applied was estimated by measuring their germination according to the bioassay described in Larena et al. (2002). After treatment, tomato seedlings treated were transplanted from seedbeds into 100 ml-flasks containing 125 ml sterile Hoagland N° 2 solution (Hoagland and Arnon, 1950), as described by Larena et al. (2007). Microconidia of FOL were added to the flasks just before transplanting, giving a final concentration of 10^5^ microconidia/ml. Control treatments were: (i) a set of plants inoculated with FOL but not treated with either wtPO212 or their transformants, (ii) a set of plants not inoculated with FOL but treated with either wtPO212 or each transformant (PO212_ar1, PO212_arRED3, PO212_in5, and PO212_inGFP9), and (iii) a set of plants not inoculated with FOL and not treated with either wtPO212 or each transformant. Five replicate flasks, each containing four plants, were used for each treatment. The flasks were placed in a randomized complete block design in a growth chamber for 4 weeks under conditions described earlier in the subsection. Each complete assay was carried out at least twice.

The density of *P. rubens* in the seedbed substrate and in the rhizosphere of three plants per treatment was estimated just before transplanting as CFU (colony forming units) per gram of dry soil and fresh root, respectively as described in Larena et al. (2003).

Every 2–4 days after transplanting, the following parameters were evaluated: consumption of nutrient solution per plant, flask and day; number of leaves per plant; and disease index in leaves according scale described in De Cal et al. (1995).

Disease severity and the area under the disease progress curve (AUDPC) over time was calculated using the disease index described below (Campbell and Madden, 1990) for each treatment. At the end of the experiment, the roots and aerial parts per flask were weighed. Then, all root plants were transferred to humid chambers and the presence or absence of mycelium of the pathogen in the crown of plants was determined after 5 days incubation at 25°C.

### Detection of dsred and GFP genes expression

Germlings were cultured in supplemented watch minimal medium (WMM plus 1% glucose (w/vol), 5 mM ammonium tartrate and 25 mM NaH_2_PO_4_) and incubated at 25°C for 16–18 h (Peñalva, 2005), using uncoated glass-bottom dishes (Mat Tek Corporation, Ashland, MA). *In vivo* imaging was performed at RT using a DMI6000B inverted microscope (Leica, Deerfield, IL) equipped with a heating insert (PeCon, Erbach, Germany), a Hamamatsu ORCA ER-II camera, an EL6000 external light source for epifluorescence excitation, an HCX 63x 1.4 NA objective, and Semrock Brightline GFP-3035B and TXRED-4040B (for RFP detection) filter sets. Images were recorded with an ORCA-ER digital camera (Hamamatsu Photonics) and processed with Metamorph (Universal Image) or ImageJ 1.37 (http://rsb.info.nih.gov/ij/) software.

For detection of fluorescent strains on tomato roots, experiments were carried out in growth chambers on tomato plants treated with an aqueous conidial suspension of each fluorescent transformed strain (PO212_arRED3 and PO212_inGFP9) in order to study the colonization of tomato roots by BCA PO212 as described in Nahalkova et al. (2008) with modifications.

Tomato seeds cv. San Pedro were surface sterilized by immersion in 1.25% sodium hypoclorite for 20 min and rinsed three times with sterile distilled water (SDW). Seeds were germinated on malt extract agar in Petri dishes at an inclination of 60° and incubated at 22°C for 4 days in the dark. Roots were dipped in the conidial suspension of PO212_arRED3 or PO212_inGFP9 (10^6^ conidia/ml) in Hoagland N°2. Since Hoagland N°2 lacks of a main carbon source to promote fungal growth, these tests were made with or without addition of sucrose (1 g/L). As non-inoculated treated control, plants were treated similarly but in sterile Hoagland solution. Three plants were examined at every time point of observation and the whole experiment was repeated twice.

Fluorescence images were acquired with an upright Eclipse 80i microscope (Nikon, Melville, NY) equipped with Brightline GFP-3035B and TXRED-4040B filter sets (Semrock, Rochester, NY), a 100-W mercury lamp epifluorescence module, a Uniblitz (Rochester, NY) external shutter, a 60x 1.40-numerical aperture (NA) plan apochromat objective, and a ORCAERG camera (Hamamatsu, Bridgewater, NJ).

Observations of the full length of each root were at 28 and 48 h after inoculation using a confocal laser scanning microscopy Leica SP2 microscope (Leica Microsystems, Wetzlar GmbH, Germany). Digital images acquired from individual channels were processed using LAS AF 2.6.0 program (Leica Microsystems CMS GmbH, Germany).

### Statistical analyses

Data on the cultural characteristics (growth rate *in vitro*, conidia germination and length of germ tubes) and the biocontrol efficacy (disease severity, AUDPC, number of leaves, consumption of nutritive solution, stem and root weight) were analyzed by one-way analysis of variance (ANOVA) using Statgraphics® Centurion XVI version 16.1.03. The Student-Newman-Keul′s multiple range test at *P* = 0.05 was used for comparison of means (Snedecor and Cochran, 1980). When the variances were not homogeneous, the significance of the differences between the means was determined using the Kruskal-Wallis test.

The AUDPC was calculated using the disease severity percentage data (Campbell and Madden, 1990):

$$\begin{array}{l} \text{AUDPC }=\;\sum_{i\;=\;1}^{n-1}\left[\frac{\left(t_{i+1}-t_{i}\right)\left(y_{i}+y_{i+1}\right)}{2}\right] \end{array}$$

where *t* is the number of days from transplanting to the end of the assay, y is the disease severity (%) for each plant and, *n* is the number of samples.

The growth rates (mm/day) were obtained from the growth data using linear regression of the linear parts of the temporal growth curves.

All assays were repeated twice. For each assay, data of the two repeats were first subjected to ANOVA. When no statistical differences were found, the data of the two repeats were combined into a single set of data. However, when statistical differences were found only the data of one repeat was shown.

## Results

### Transformation of PO212 with integrative and autoreplicative plasmids by pyrimidine auxotrophy complementation

To approach genetic transformation of the BCA PO212, we used the standardized procedure for transformation of *A. nidulans* by using PEG-calcium-competent protoplasts (Tilburn et al., 1983). Using mixtures of cell wall lysing enzymes, Vinoflow or Glucanex-100G, protoplasts of wtPO212 were prepared as described previously for *A. nidulans*. Protoplasts were viable after the transformation procedure without addition of DNA and formed colonies on regeneration medium containing 1M sucrose as osmotic stabilizer.

Next, we approached selective transformation of PO212 by taking advantage of previous isolation and characterization of a pyrimidine auxotroph, the PO212_18.2 strain (Villarino et al., 2016) carrying a mutation truncating PyrG at amino acid 104 that we designate now as *pyrG1* allele. In this study, PO212_18.2 was used as recipient for transformation of two different types of plasmids: autoreplicative and integrative.

We analyzed complementation of the *pyrG1* mutation by using homologs from *N. crassa, pyr*-4 (*pyr4*^*Nc*^) and *pyrG* from *A. fumigatus, pyrG*^*Af*^. To test complementation by *pyr4*^*Nc*^ we used the autoreplicative plasmid pRG3-NotI-AMA1 largely used in *A. nidulans* (Osherov et al., 2000), and for *pyrG*^*Af*^ we used a plasmid constructed to generate chimeric-fusion proteins in *A. nidulans*, pFNO3 (a kind gift of S. Osmani; Nayak et al., 2006). To introduce these plasmids into PO212_18.2 we produced protoplasts as described above. PO212_18.2 generated abundant protoplasts when digested with Glucanex-100G or Vinoflow and were competent for PEG-calcium mediated transformation, obtaining >200 transformants per μg of autoreplicative plasmid and ten to five times less for a μg of the integrative plasmid. At least 20 of these transformants purified to homokaryosis by successive replica-plating and all formed stable colonies along the purification process. These results showed that either *pyr4*^*Nc*^ or *pyrG*^*Af*^ were able to complement the *pyrG1* mutation in PO212_18.2.

**Figure 1:**
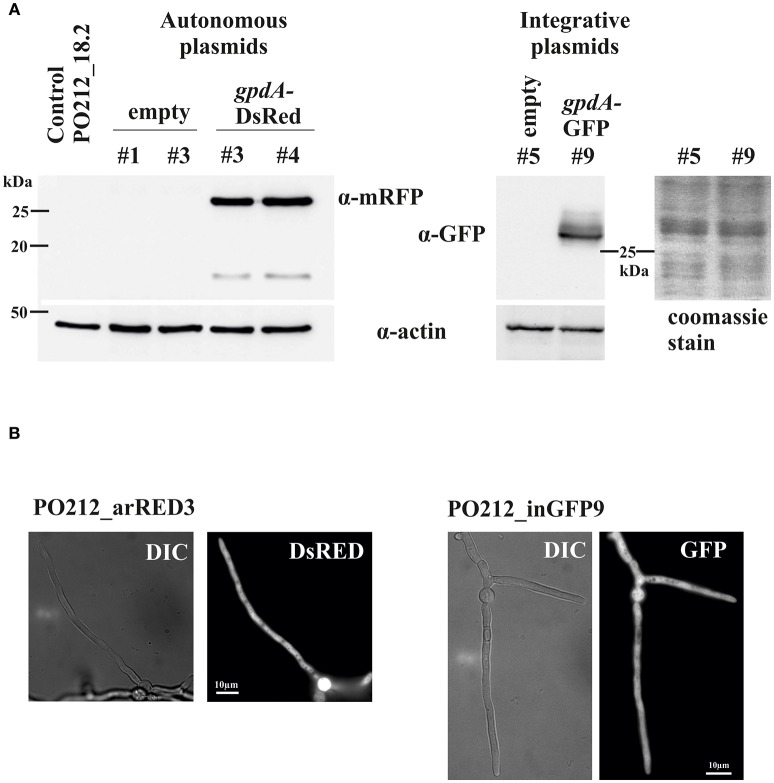
Detection of fluorescent proteins in PO212. **(A)** Western-blot analysis of two transformants expressing the DsRed and GFP fluorescent proteins. An antibody against mRFP was used for visualization of DsRed and an specific antibody against GFP was used. As control protein extracts from recipient strain PO212_18.2 and transformants carrying an empty autonomous replicating and integrative plasmids were included. Detection of actin was used as loading control. Also a coomassie stain of SDS-PAGE of PO212_in5 and PO212_inGFP9 protein extracts in the region of GFP detection is shown. **(B)** Fluorescence detection in cells of transformants expressing either GFP (PO212_inGFP9) or DsRed (PO212_arRED3). DIC, Nomarski interference optics. Scale bars are 10 μm.

Subsequently we addressed the generation of strains of PO212 that could express green or red fluorescent proteins. For this purpose we transformed plasmids in PO212_18.2 that would express GFP and DsRed under the constitutive *A. nidulans* glyceraldehyde-3-phosphate dehydrogenase promoter (*gpdA*^*p*^) shown in Figure S1. As described before for the empty plasmids, we were able to obtain transformants with both the integrative plasmid containing *GFP* gene, and the autonomous replicating plasmid carrying the Ds-Red Express coding gene. Transformants carrying either construct were isolated, purified to homokaryosis and tested for stability. PCR and Western blot analyses of transformants confirmed that the *GFP* and *Ds-Red Express* genes were present in these strains and that all selected transformants expressed the fluorescent protein and showed to be fluorescent in WMM (Figure 1A). GFP and DsRed regularly distributed along the cytoplasm (Figure 1B).

Transformants showed the same colony and cellular morphologies as the parental PO212_18.2 strain. Phenotypic differences of transformants with respect to the parental strain were limited to the complementation of pyrimidine auxotrophy. Among the collection, four transformants containing either empty or fluorescent expressing vectors, being integrative or autoreplicative plasmids were selected for subsequent ecophysiological characterization, biocontrol efficacy, and microscopy assessments.

### Ecophysiological characterization of transformed strains

Growth rates, conidia germination and length of germ tubes of PO212_ar1, PO212_arRED3, PO212_in5 and PO212_inGFP9 strains at different pH and temperatures compared to those previously described for the wtPO212.

#### Growth studies in vitro

The growth rate (mm/day) of the PO212 transformed strains in PDA at different temperatures and pH values showed the same trend compared to wtPO212 (Figures S2, S3). The largest growth was observed at 25°C, giving the lowest values at 4 and 35°C for all strains (Tables 1, 2). The growth rate of all strains was lower at pH 4 than to the rest of pH, showing the same growth for all strains, at pH 5.5, 7, and 8 (Tables 1, 2). In general, wtPO212 was the one that showed the greatest growth for all temperatures and pH (Tables 1, 2).

**Table 1:** Comparison of the colony growth rate (mm/day) of the wild-type PO212 (wtPO212) and the transformed PO212 strains (PO212_ar1, PO212_arRED3 and PO212_in5) at different temperatures and pH values growing on potato dextrose agar.

	**Temperature (**^**°**^**C)**				**pH**			
	**4**	**15**	**25**	**35**	**4**	**5.5**	**7**	**8**
wtPO212	1.4b	3.7a	5.4a	1.6a	4.4a	5.4a	5.5a	5.3a
PO212_ar1	1.6a	3.0c	4.5c	1.1b	3.7c	4.5c	4.3d	3.4b
PO212_arRED3	1.1c	3.4b	5.2b	1.1b	4.1b	5.2b	5.2c	5.1a
PO212_in5	1.55ab	3.6a	5.3b	1.5a	4.4a	5.3b	5.3b	5.1a
MS_within_	9.4 × 10^−3^		4.5 × 10^−3^	2.1 × 10^−2^		4.5 × 10^−3^		
K-W test		*P* = 6.9 × 10^−7^			*P* = 3.1 × 10^−6^		*P* = 3.9 × 10^−7^	*P* = 3 × 10^−7^

*Rate was determined as the slope of the linear portion of a curve produced by plotting growth diameter (mm) vs. time (days). Data are displayed as the mean of 10 replications. In each column, values followed by the same letter are not significantly different (P ≤ 0.05) according to Student-Newman-Keul's range test or Kruskal-Wallis (K-W) test. The repeat confirmed the results, so only results from one repeat are shown. MS_within_ — error mean square*.

**Table 2:** Comparison of the colony growth rate (mm/day) of the wild-type PO212 (wtPO212) and the transformed PO212 strain, PO212_inGFP9, at different temperatures and pH values growing on potato dextrose agar.

**Strain**	**Temperature (**^**°**^**C)**				**pH**			
	**4**	**15**	**25**	**35**	**4**	**5.5**	**7**	**8**
wtPO212	0.6a	3.5a	5.6a	0.4a	4.1a	5.2a	5.0a	4.8a
PO212_inGFP9	0.6a	3.2b	4.3b	0.4a	3.3b	3.7b	3.9b	3.9b
MS_within_	7.4 × 10^−4^	6.5 × 10^−2^	0.1	1.2 × 10^−3^	7.6 × 10^−2^	0.1	6.0 × 10^−2^	0.2

*Rate was determined as the slope of the linear portion of a curve produced by plotting growth diameter (mm) vs. time (days). Data are displayed as the mean of 10 replications. In each column, values followed by the same letter are not significantly different (P ≤ 0.05) according to Student-Newman-Keul's range test or Kruskal-Wallis (K-W) test. The repeat confirmed the results, so only results from one repeat are shown. MS_within_ — error mean square*.

#### Conidia germination and length of germ tubes studies in vitro

Effective germination of conidia was measured. At all tested temperatures and pH values, the complete set of transformed strains showed similar rates of germination in CDB compared to that of wtPO212 (Tables S1, S2). In all cases, differences were not significant (*P* ≤ 0.05) except for PO212_arRED3 (64%) at pH 4 (Table S1) which showed a percentage of germination significantly (*P* ≤ 0.05) lower than wtPO212 (81.3%). While PO212_inGFP9 (27.4%) at pH 8 shown a percentage of germination significantly (*P* ≤ 0.05) higher than wtPO212 (13.8%) (Table S2). At 4°C conidia from any strain were unable to germinate, while at 15°C (0.4–11%) and 35°C (0.1-35%) either conidia did not germinate or cultures showed a low rate of germination (Tables S1, S2). The highest percentage of germination measured at 25°C (in a range between 63.8 and 77.9%) (Tables S1, S2). While all the percentage of germination were high and similar to all the pH tested (Tables S1, S2).

The length of germ tubes was similar for the wtPO212 and all transformed strains at all tested temperatures and non-significant (*P* ≤ 0.05) differences were reported between the all transformed strains and wtPO212 (Tables S3, S4). The length of germ tubes showed differences significant (*P* ≤ 0.05) between wtPO212 (36.5 μm) and PO212_ar1 (30.0 μm) at pH 4, and between wtPO212 (36.5 μm) and PO212_arRED3 (29.9 μm) and PO212_in5 (22.4 μm) at pH 8 (Table S3). In all strains, the greatest length of germ tubes was given at pH 5.5 (in a range between 28.1 and 27.7 μm) and 25°C (in a range between 20.9 and 48.6 μm) (Tables S3, S4).

### Biocontrol efficacy assays on tomato plants

To test the possible effect of random integration or autonomous maintenance of transforming DNA, both types of PO212 transformants were analyzed for their effectiveness against FOL on tomato plants in growth chamber.

Previous to performing biocontrol assays, we verified that assayed *Penicillium* strains showed a similar percentage of conidia viability at application time and was higher than 80%. Additionally, populations of PO212 strains estimated just before transplanting to flasks were similar in the roots and in the seedbed substrate, 10^5^-10^6^ CFU/g fresh root weight (g dry substrate weight). These controls ensured a proper treatment of plants with *Penicillium* strains. We also verified that plants not subjected to inoculation with the pathogen, did not display any disease symptoms, when treated or not (Tables 3, 4) with any of the PO212 strains. And, that wtPO212, PO212_ar1, PO212_arRED3, PO212_in5, and PO212_inGFP9 were not detected either in the rhizosphere or in the substrate of the seedlings of untreated plants.

**Table 3:** Effect of the wild-type PO212 (wtPO212) and the transformed strains (PO212_ar1, PO212_arRED3, and PO212_in5) on disease severity, and AUDPC caused by *Fusarium oxysporum* f. sp. *lycopersici* (FOL), and nutritive solution consumption, leaves number, stem and root weight on tomato plants cv. San Pedro at 13 days after their transplanting and inoculating with pathogen.

**Strain**	**% Disease severity**	**AUDPC**	**Consumption (ml/plant/day)**	**Leaves number**	**Stem weight (g)**	**Root weight (g)**
wtPO212	40.0b	264.7b	0.7a	5.8a	3.3a	4.8a
PO212_ar1	42.5b	281.3b	0.3b	5.2b	2.3ab	4.5a
PO212_arRED3	40.6b	326.3b	0.2bc	5.3b	2.0bc	3.7ab
PO212_in5	30.6b	228.8b	0.4b	5.4ab	3.9a	4.7a
Untreated	70.3a	541.4a	0.1c	4.4c	1.2c	3.3b
MS_within_	114	5031.4	0.1	0.1	0.4	0.5

*Data are the mean of five replicates with four plants per replicate. In each column, values followed by the same letter are not significantly different (P ≤ 0.05) according to Student-Newman-Keul's range test. AUDPC is the area under disease progress curve estimated as described in Campbell and Madden (1990). The repeat confirmed the results, so only results from one repeat are shown. Untreated: plants treated with sterile distilled water, instead of any strain of PO212 and inoculated with FOL. MS_within_ — error mean square*.

**Table 4:** Effect of the wild-type PO212 (wtPO212) and the transformed strain PO212_inGFP9 on disease severity and AUDPC caused by *Fusarium oxysporum* f. sp *lycopersici* (FOL), and nutritive solution consumption, leaves number, stem, and root weight on tomato plants cv. San Pedro at 14 days after their transplanting and inoculating with pathogen.

**Strain**	**% Disease severity**	**AUDPC**	**Consumption (ml/plant/day)**	**Leaves number**	**Stem weight (g)**	**Root weight (g)**
wtPO212	44.4b	305.6 ab	1.8b	4.6a	5.1b	6.8b
PO212_inGFP9	35.6b	225.6b	2.8a	5.8a	9.7a	10.8a
Untreated	61.3a	335.9a	1.6b	4.6a	5.3b	6.8b
MS_within_	134.4	3818.4	0.4	0.6	3.7	2.2

*Data are the mean of five replicates with four plants per replicate. In each column, values followed by the same letter are not significantly different (P ≤ 0.05) according to Student-Newman-Keul's range test. AUDPC is the area under disease progress curve estimated as described in Campbell and Madden (1990). The repeat confirmed the results, so only results from one repeat are shown. Untreated: plants treated with sterile distilled water, instead of any strain of PO212 and inoculated with FOL. MS_within_ — error mean square*.

The disease severity on the untreated and inoculated plants with FOL was 70.3% in assay 1 (Table 3), and 61.3% in assay 2 (Table 4). Plants pretreated with of each of the PO212 strains showed a significant (*P* ≥ 0.05) reduction of disease severity (28–56% of reduction), and AUDPC (10 and 58% of reduction) compared to those non-treated and inoculated plants (control treatment) (Tables 3, 4). No significant differences (*P* ≥ 0.05) in these parameters were observed between the treatments with wtPO212 and those using each of the transformed strains (assay 1 Table 3, assay 2 Table 4).

All transformed strains increased significantly (*P* ≥ 0.05) the nutritive solution consumption, leaves number and stem and root weight in infected plants when compared to its respective untreated control (Tables 3, 4), except the consumption, the stem and root weight for PO212_arRED3 (Table 3). It is worth noting that we generally use all these parameters to evaluate the efficacy of PO212 as BCA against the pathogen. Greater variability in parameters such as the solution consumption, leaves number and stem and root weight could be a consequence of the higher effect of other variables in addition to the pathogen than in the indicative parameters of the disease (severity and AUDPC).

### Detection of DsReD and GFP expression in liquid medium and tomato roots

All transformants displayed DsRed and GFP expression when were cultured in supplemented WMM and observed with an epifluorescence microscope. Nevertheless, variation of fluorescent levels was observed probably due differences in expression of fluorescent proteins among the transformants. Transformants expressing DsRed and GFP displayed dispersed and homogeneous fluorescence along vegetative cells and reproductive forms (asexual spores) of PO212 (Figure 1B). Only vacuoles were empty of fluorescence and appeared as dark round areas of various sizes within the cytoplasm. Photobleaching was not appreciable during microscopy analyses.

**Figure 2:**
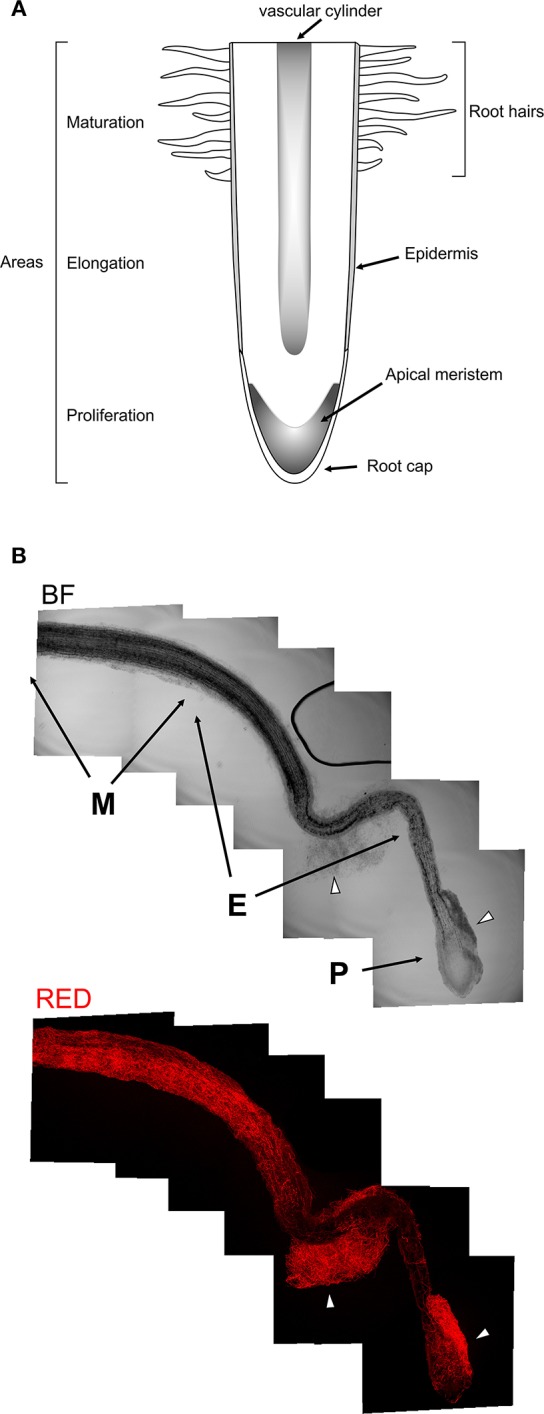
Colonization of tomato roots by PO212_arRED3. **(A)** Schematic representation of a root. Indicated are the main regions and structures present in a root. **(B)** Brigth field (BF) montage of series of images covering a main tomato root. Below is the same root visualized by confocal microscopy for DsRed fluorescence. M, E, and P indicate maturation, elongation and proliferation areas, respectively. Arrowheads point to mycelial accumulations on the root surface.

**Figure 3:**
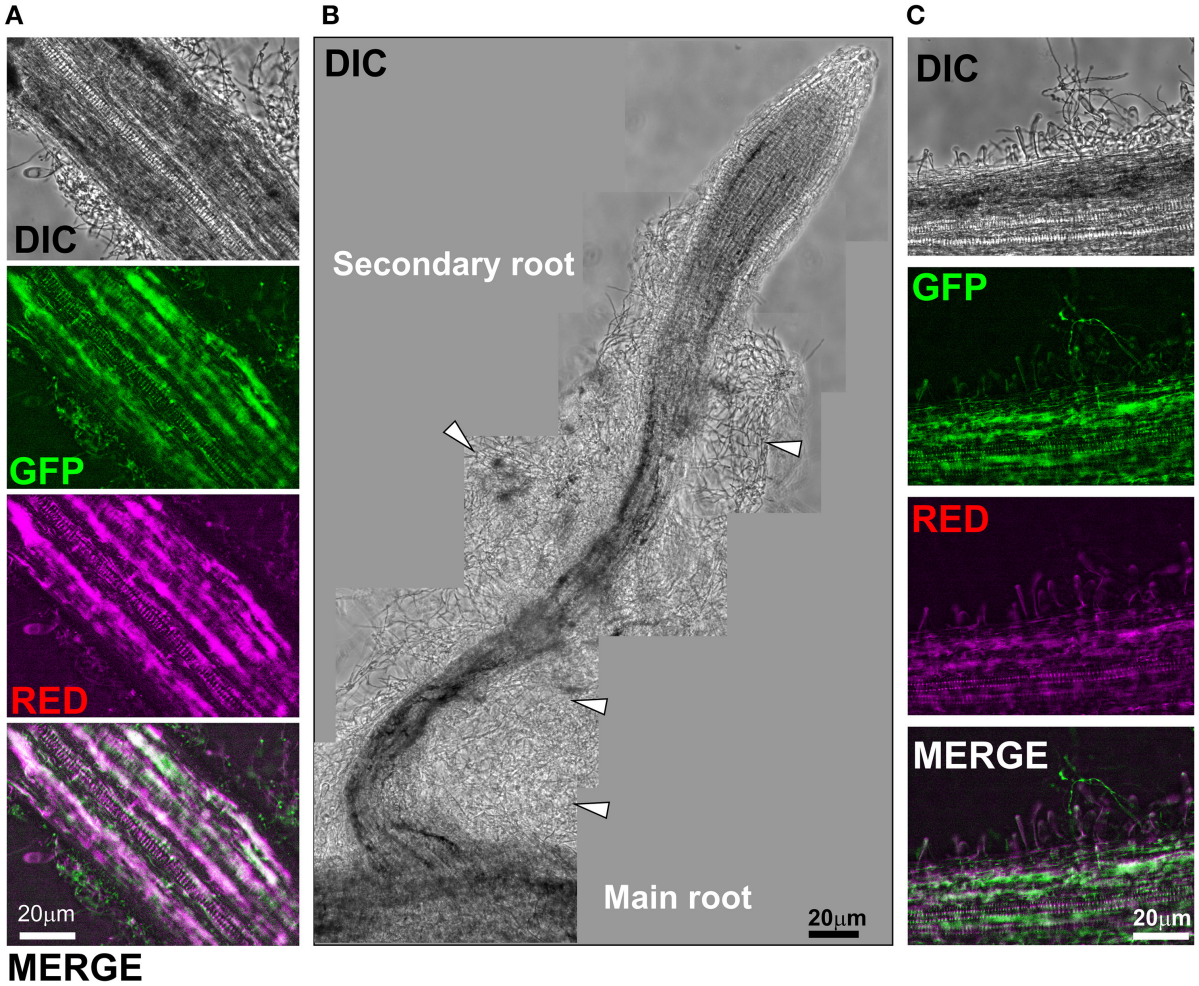
Colonization of tomato roots by PO212 expressing GFP. Tomato roots were treated with 10^6^ spores/ml. PO212_inGFP9 grew for 28 h in Hoagland containing 0.1% sucrose. **(A)** Shows a section of main root colonized by PO212. GFP channel illuminates fungus and root. Red channel (magenta) illuminates root. **(B)** Bright field image montage of a secondary root colonized by PO212. Arrowheads point to mycelial masses. **(C)** Magnification of main root surface. Root hairs were visible and fungus growing intermingled. Magnifications for all panels are 200x. Scale bars are 20 μm.

**Figure 4:**
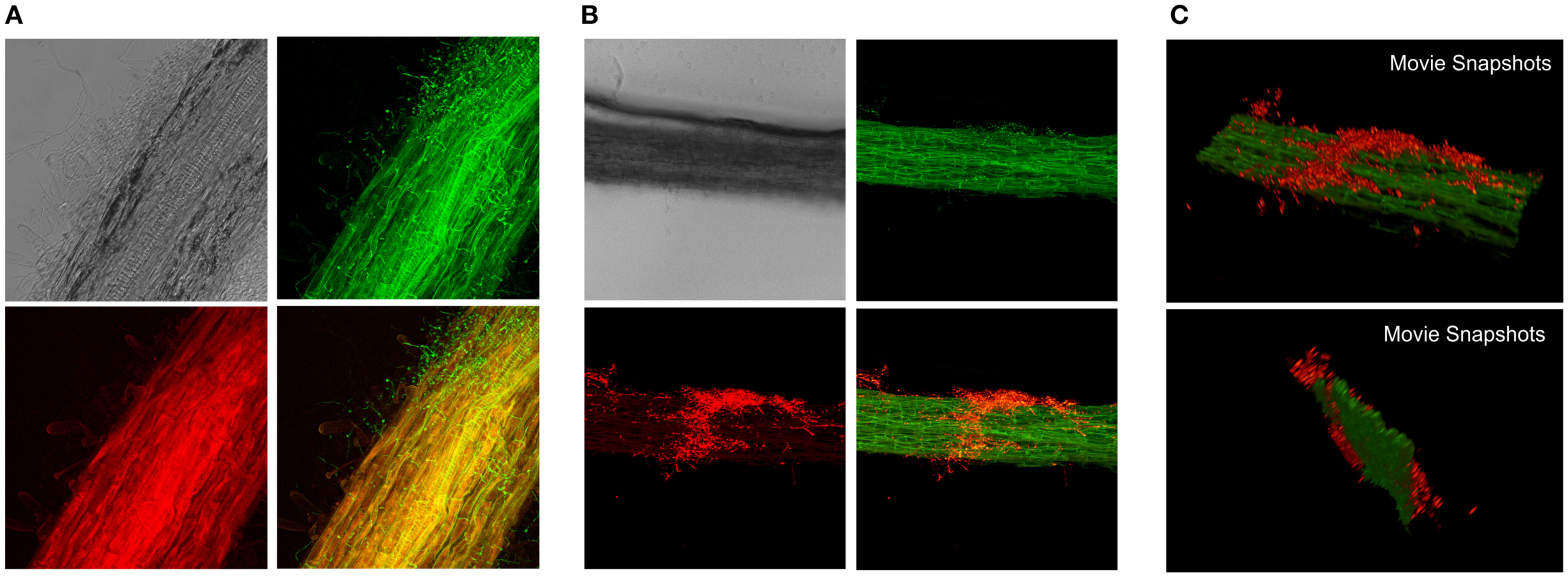
Confocal imaging of tomato roots colonization by fluorescent PO212 strains. Maximal projections of tomato root sections colonized by **(A)** green and **(B,C)** red fluorescent PO212 strains. Mycelia were visualized on the surface of tomato roots. **(A)** Is composed of images from Nomarski optics (top left), green channel for visualization of GFP (top right), red channel for visualization of root autofluorescence (bottom left) and the merged image of green and red channels. **(B)** Is composed of images from Nomarski optics (top left), green channel for visualization of root autofluorescence (top right), red channel for visualization of DsRed fluorescence (bottom left) and the merged image of green and red channels. **(C)** Two snapshots of Movie S1 showing the 3D reconstruction of a colonized root section.

Following microscopic characterization, we selected one transformant expressing each of the fluorescent proteins optimally for microscopic analyses. PO212 transformed strains PO212_arRED3 and PO212_inGFP9 were used for further studies to determine the mode of BCA-plant interaction during colonization of tomato roots. The *in situ* observation studies were performed either using epifluorescence or confocal microscopy. Tomato seeds were germinated on solid media for 4 days and seedlings were submerged into Hoagland's solution plus sucrose and a suspension of conidiospores from GFP and DsRed expressing transformants was added. After 28 and 48 h fungal growth was evaluated under the microscope. The location of fungal cells was restricted to the root system (Figures 2, 3). Transformed strains did not colonize stems of leaves (data not shown). We observed fungal growth along surface of main root and also on secondary roots (see Figure 2A for a schematic representation of a root, Figure 3). Mycelial accumulations were observed mainly at the elongation zone of primary and secondary roots (white arrowheads Figures 2B, 3B). Most of roots lacked of massive fungal colonization at the root cap, although this was highly variable (compare Figures 2B, 3B). It was of note the high autofluorescence of tomato roots in both green and red channels. For epifluorescence imaging, merging GFP and RED images allowed identification of PO212_inGFP9 cells on the surface of roots (Figures 3A,C), this strategy was also used when confocal visualization (Figure 4A). In contrast, fluorescence levels of PO212_arRED3 were optimal for confocal imaging (Figures 2B, 4B). In both analyses we verified that fluorescent transformed strains did not penetrate the vascular system. Xylem and phloem vessels were not colonized by PO212 (see Figures 2, 4 and Movie 1). In contrast fungal cells remained well adhered to the root epidermis not interfering with production of root hairs (Figures 3C, 4A).

## Discussion

A major objective of this work was to obtain fluorescent protein-tagged PO212 in order to study host-pathogen-PO212 interactions. Our report describes the transformation of *P. rubens* strain 212, an important BCA against several horticultural diseases, with Ds-Red and GFP and the use of transformed strains to study the tomato colonization pattern by this BCA.

Our results show that PO212 is competent for transformation via protoplasts and mediated by calcium and PEG. We successfully obtained protoplasts and they were transformed using a positive selection system based on complementation of mutations causing pyrimidine auxotrophy (Bañuelos et al., 2003). Complementation of loss-of-function mutations in the gene coding for orotidine 5'-phosphate decarboxylase has become a convenient procedure to avoid the use of antibiotics in selection procedures (Fierro et al., 2004). Work in *P. nalgiovense* also showed that the availability of a *pyrG* mutant strain and a transformation system based on uridine auxotrophy complementation with a homologous *pyrG* gene offers several advantages over transformation based on antibiotic (phleomycin) resistance (Fierro et al., 2004). So, firstly the transformation efficiency is much higher when using the *pyrG* gene than with either of the other systems; and secondly the use of the homologous *pyrG* gene as selection marker allows counter-selection using 5-FOA, which is very useful to perform successive transformations of the same strain (d′Enfert, 1996; Fierro et al., 2004). Finally, any construction can be introduced in a single copy at the *pyrG* locus by gene targeting using a mutant *pyrG* gene (containing a point mutation) in the transforming vector (Gouka et al., 1991; Kosalková et al., 2000).

In view of our success in transforming PO212, we approached the heterologous expression of widely used fluorescent proteins. We have expressed by means of two vectors, integrative and autoreplicative, the fluorescent proteins GFP and DsRed-Express, using the constitutive promoter of the *glyceraldehyde 3P dehydrogenase* of *A. nidulans*, which has been used successfully for the expression of these fluorescent proteins in other ascomycete fungi (Mikkelsen et al., 2003). Some strains of *P. chrysogenum* have been subjected to transformation procedures with integrative (Cantoral et al., 1993) or autonomous replicating (Fierro et al., 1996) plasmids and co-transformation (Bañuelos et al., 2003). Here we show that integrative plasmids can be used in PO212, however, as observed in other Penicillia, the number of transformants is reduced with this type of DNA vectors (Bañuelos et al., 2003; Fierro et al., 2004). The use of autonomously replicating vectors has been of great interest. As in *P. nalgiovense*, the transformation efficiency achieved with autonomous-replicating vectors make them excellent tools for constructing new tools to understand key processes in fungi of industrial interest (Fierro et al., 2004). This can be also the case for the BCA PO212 for constructing genomic libraries to clone genes by complementation, which will help the molecular studies on pathways involved in biocontrol in this fungus. In addition, the possibility of manipulating a strain without affecting its genome integrity is important to prevent undesirable crossed effect of vector integration, becoming of greatest interest when using an isolate as PO212.

Both integrative and autonomous replicating plasmids allow the expression of proteins of interest in the recipient strains. In this work, we show the expression in PO212 of two useful tools for microscopy, sGFP and DsRed-Express proteins. Importantly, microscopic analysis of the transformants in liquid medium revealed homogeneity of the fluorescent signals proving that the use of green or red fluorescent proteins is a convenient, fast, and effective approach to label PO212 strain for studying plant–fungus interactions.

Our next step was to demonstrate that integrative or autorreplicate vectors and expression of either GFP or DsRed did not alter the biocontrol capacity and culture characteristics of PO212 for further microscopic studies of root colonization. As has been described in other BCAs, such *Trichoderma harzianum*, it is essential to compare the physiological traits and biocontrol ability of the obtained transformants with wild type strains before carrying out time-consuming ecological studies because transformed strains must be able to maintain their biocontrol activity (Lo et al., 1998). Thus transformants of PO212 showed a phenotype practically indistinguishable from that of the wtPO212 with respect to growth rate, colony morphology, and biocontrol efficacy on tomato. As described for the biocontrol strain, *T. atroviride* strain P1, whose transformation with the *gfp* gene did not affect its biocontrol ability (Lu et al., 2004), our DsRed and GFP-expressing PO212 transformants are effective against Fusarium wilt in tomato plants, reaching levels similar to wtPO212. These biocontrol efficacy levels are consistent with those obtained in previous studies conducted with this BCA reaching up to 80% (De Cal et al., 1995, 1997, 2000).

The efficiency of biological control of plant diseases is strongly influenced by the complexity of the tripartite interaction between plant, BCA and pathogen and their visualization studies in the rhizosphere contribute to a better understanding of these interactions (Bolwerk and Lugtenberg, 2005). For this reason, our first approach to these studies has been the labeling of our biocontrol strain with fluorescent proteins and their visualization in the tomato rhizosphere. To our knowledge, reports on visualization of root colonization by BCA fungal strains with reporter or marker genes for detection and monitoring of introduced BCA once applied are scarce (Green and Jensen, 1995; Lo et al., 1998; Lübeck et al., 2002). Interaction of BCA-plant-pathogen needs of adequate microscopic tools to simultaneously visualize *in situ* relations amongst these three components. Combination of fluorescent-tagged proteins and advances in microscopy improve monitoring of growth and dispersion of BCA and/or pathogen along the host plant. These techniques have been used in many colonization studies (Nahalkova et al., 2008) but have been veiled for PO212 until this work. PO212 remained at the root system, associated to the root surface, with no signal in the xylem, neither in stem or leaves. These observations confirmed conclusions of previous studies using standard microscopy (De Cal et al., 2000).

As a summary of everything discussed above we can indicate that transformed PO212 strains showed to maintain the properties of PO212 as BCA, hence opening a new way for future modifications in this strain dedicated to decipher the mechanisms underlying biocontrol. We show the usefulness of pyrimidine prototrophy for the study of BCA-plant interaction, avoiding the use of antibiotics and other chemicals to maintain vectors into the fungus. The possibility to use autoreplicative vectors in PO212, also during biocontrol experiments, will facilitate a rapid way to obtain transformants allowing the expression of genes and regulators for their evaluations in this process. In the case of green or red fluorescent labeled PO212 strains, these can be used in the laboratory to score how PO212 interact with other fungi isolated from soil or plant.

## Author contributions

MV performed experimental work, analyzed data, wrote manuscript. EE performed experimental work, analyzed data, wrote manuscript. PM wrote manuscript. IL analyzed data, wrote manuscript.

### Conflict of interest statement

The authors declare that the research was conducted in the absence of any commercial or financial relationships that could be construed as a potential conflict of interest.

## Supplementary material

Figure S1 Schematic representation of plasmids used in this work. Autoreplicative plasmids have a pUC19 backbone, and integrative plasmids are of TOPO series (pCRII-TOPO). AMA1 indicate presence of autonomous replicative sequences. As selection markers for *P. rubens* transformation, plasmids carry either *pyr*-*4* or *pyrG* genes from *N. crassa* and *A. fumigatus*, respectively. Indicated are the presence of chimaeric transgenes: GA5xGFP, without promoter or *gpdA*^*p*^ (glyceraldehyde 3-P dehydrogenase promoter). DsRed was expressed under *gpdA*^*p*^ and also carries the terminator from *trpC* gene, *trpC*^*ter*^.

Figure S2 Effect of temperature and pH on the radial growth of wild-type PO212 and the transformed PO212 strains. Diameter of colonies grown at the indicated temperatures and pH values on PDA are represented along 14 days of incubation. Values are the means of 10 replications. Standard errors are shown. Strains code: PO212, PO212_ar1, PO212_arRED3, PO212_in 5 .

Figure S3 Effect of temperature and pH on the radial growth of wild-type PO212 and the transformed PO212 strain PO212_inGFP9. Diameter of colonies grown at the indicated temperatures and pH values on PDA are represented along 14 days of incubation. Values are the means of 10 replications. Standard errors are shown. Strains code: PO212, PO212_inGFP9.

Table S1 Comparison of the germination percentage (%) of the wild-type PO212 (wtPO212) and the transformed PO212 strains (PO212_ar1, PO212_arRED3, and PO212_in5) at different temperature (25 and 35°C) and pH (4, 5.5, 7, and 8).

Table S2 Comparison of the germination percentage (%) of the wild-type PO212 (wtPO212) and the transformed PO212 strain, PO212_inGFP9 at different temperatures and pH values.

Table S3 Comparison of the length of the germ tube (μm) of the wild-type PO212 (wtPO212) and the transformed PO212 strains (PO212_ar1, PO212_arRED3, and PO212_in5) at different temperatures and pH values.

Table S4 Comparison of the length of the germ tube (μm) of the wild-type PO212 (wtPO212) and the transformed PO212 strain, PO212_inGFP9 at different temperatures and pH values.

Movie 1 3D reconstruction of a z-stack from a section of a tomato root (green) colonized by PO212_arRED3 (red). Movie is shown at 15 frames per second.

## References

[B1] Bae Y. S. Knudsen G. R. (2000). Cotransformation of *Trichoderma harzianum* with ß-Glucuronidase and green fluorescent protein genes provides a useful tool for monitoring fungal growth and activity in natural soils. Appl. Environ. Microbiol. 66, 810–815. 10.1128/AEM.66.2.810-815.2000 10653755 PMC91900

[B2] Bañuelos O. Naranjo L. Casqueiro J. Gutierrez S. Martin J. F. (2003). Co-transformation with autonomous replicating and integrative plasmids in *Penicillium chrysogenum* is highly efficient and leads in some cases to rescue of the intact integrative plasmid. Fungal Genet. Biol. 40, 83–92. 10.1016/S1087-1845(03)00081-1 14516761

[B3] Beri R. K. Turner G. (1987). Transformation of *Penicillium chrysogenum* using the *Aspergillus nidulans amdS* gene as a dominant selective marker. Curr. Genet. 11, 639–641. 10.1007/BF00393928 3131026

[B4] Bhuiyan S. A. Ryley M. J. Galea V. J. Tay D. (2003). Evaluation of potential biocontrol agents against *Claviceps africana in vitro* and *in vivo*. Plant Pathol. 52, 60–67. 10.1046/j.1365-3059.2003.00799.x

[B5] Bolwerk A. Lagopodi A. L. Lugtenberg B. J. J. Bloemberg G. V. (2005). Visualization of interactions between a pathogenic and a beneficial *Fusarium* strain during biocontrol of tomato foot and root rot. MPMI 18, 710–721. 10.1094/MPMI-18-0710 16042017

[B6] Bolwerk A. Lugtenberg B. J. J. (2005). Visualization of interactions of microbial biocontrol agents and phytopathogenic fungus *Fusarium Oxysporum* F. Sp. *Radicis-Lycopersici* on tomato roots, in PGPR: Biocontrol and Biofertilization, ed Siddiqui Z. A. (Dordrecht: Springer), 217–231.

[B7] Bull J. H. Smith D. J. Turner G. (1988). Transformation of *Penicillium chrysogenum* with a dominant selectable marker. Curr. Genet. 13, 382. 10.1007/BF00365658 3135949

[B8] Buron-Moles G. López-Pérez M. González-Candelas L. Viñas I. Teixidó N. Usall J.. (2012). Use of GFP-tagged strains of *Penicillium digitatum* and *Penicillium expansum* to study host-pathogen interactions in oranges and apples. Int. J. Food Microbiol. 160, 162–170. 10.1016/j.ijfoodmicro.2012.10.005 23177056

[B9] Campbell C. L. Madden L. V. (1990). Introduction to Plant Disease Epidemiology. 1990. New York, NY:John Wiley & Sons.

[B10] Cantoral J. M. Gutierrez S. Fierro F. Gil-Espinosa S. van Liempt H. Martin J. F. (1993). Biochemical characterization and molecular genetics of nine mutants of *Penicillium chrysogenum* impaired in penicillin biosynthesis. *J. Biol*. Chem. 268, 737–744. 8416976

[B11] Cove D. J. (1966). The induction and repression of nitrate reductase in the fungus *Aspergillus nidulans*. Biochim. Biophys. Acta 113, 51–56. 10.1016/S0926-6593(66)80120-0 5940632

[B12] De Cal A. Garcia-Lepe R. Melgarejo P. (2000). Induced resistance by penicillium oxalicum against *Fusarium oxysporum* f. sp. lycopersici: histological studies of infected and induced tomato stems. Phytopathology 90, 260–268. 10.1094/PHYTO.2000.90.3.260 18944618

[B13] De Cal A. Larena I. Linan M. Torres R. Lamarca N. Usall J.. (2009). Population dynamics of *Epicoccum nigrum*, a biocontrol agent against brown rot in stone fruit. J. Appl. Microbiol. 106, 592–605. 10.1111/j.1365-2672.2008.04030.x 19200324

[B14] De Cal A. Pascual S. Larena I. Melgarejo P. (1995). Biological control of *Fusarium oxysporum* f. sp. *lycopersici*. Plant Pathol. 44, 909–917. 10.1111/j.1365-3059.1995.tb02750.x

[B15] De Cal A. Pascual S. Melgarejo P. (1997). A rapid laboratory method for assessing the biological control potential of *Penicillium oxalicum* against Fusarium wilt of tomato. Plant Pathol. 46, 699–707. 10.1046/j.1365-3059.1997.d01-55.x

[B16] De Cal A. Redondo C. Sztejnberg A. Melgarejo P. (2008). Biocontrol of powdery mildew by *Penicillium oxalicum* in open-field nurseries of strawberries. Biol. Control 47, 103–107. 10.1016/j.biocontrol.2008.07.010

[B17] d′Enfert C. (1996). Selection of multiple disruption events in *Aspergillus fumigatus* using the orotidine-5'-decarboxylase gene, pyrG, as a unique transformation marker. Curr. Genet. 30, 76–82. 10.1007/s002940050103 8662213

[B18] Deng H. Gao R. Liao X. Cai Y. (2017). CRISPR system in filamentous fungi: current achievements and future directions. Gene 627, 212–221. 10.1016/j.gene.2017.06.019 28625564

[B19] Díez B. Alvarez E. Cantoral J. M. Barredo J. L. Martín J. F. (1987). Selection and characterization of *pyrG* mutants of *Penicillium chrysogenum* lacking orotidine-5'-phosphate decarboxylase and complementation by the *pyr4* gene of *Neurospora crassa*. Curr. Genet. 12, 277–282. 10.1007/BF00435290

[B20] Dong H. Z. Cohen Y. (2001). Extracts of killed *Penicillium chrysogenum* induce resistance against Fusarium wilt of melon. Phytoparasitica 29, 421–430. 10.1007/BF02981861

[B21] Dong H. Z. Cohen Y. (2002). Dry mycelium of *Penicillium chrysogenum i*nduces resistance against verticillium wilt and enhances growth of cotton plants. Phytoparasitica 30, 147–157. 10.1007/BF02979697

[B22] Etxebeste O. Herrero-Garcia E. Araujo-Bazan L. Rodriguez-Urra A. B. Garzia A. Ugalde U.. (2009). The bZIP-type transcription factor FlbB regulates distinct morphogenetic stages of colony formation in *Aspergillus nidulans*. Mol. Microbiol. 73, 775–789. 10.1111/j.1365-2958.2009.06804.x 19656299

[B23] Fang J. G. Tsao P. H. (1995). Efficacy of *Penicillium funiculosum* as a biological control agent against *Phytophthora* root rots of azalea and citrus. Phytopathology 85, 871–878. 10.1094/Phyto-85-871

[B24] Fierro F. Kosalkova K. Gutierrez S. Martin J. F. (1996). Autonomously replicating plasmids carrying the AMA1 region in *Penicillium chrysogenum*. Curr. Genet. 29, 482–489. 10.1007/BF02221518 8625429

[B25] Fierro F. Laich F. Garcia-Rico R. O. Martin J. F. (2004). High efficiency transformation of *Penicillium nalgiovense* with integrative and autonomously replicating plasmids. Int. J. Food Microbiol. 90, 237–248. 10.1016/S0168-1605(03)00306-4 14698104

[B26] Gouka R. J. van H. W. Bovenberg R. A. van den Hondel C. A. van Gorcom R. F. (1991). Cloning of the nitrate-nitrite reductase gene cluster of *Penicillium chrysogenum* and use of the *niaD* gene as a homologous selection marker. J. Biotechnol. 20, 189–199. 10.1016/0168-1656(91)90227-M 1367546

[B27] Green H. Jensen D. F. (1995). A tool for monitoring Trichoderma harzianum. II. The use of a GUS transformant for ecological studies in the rhizosphere. Phytopathology 85, 1436–1440. 10.1094/Phyto-85-1436

[B28] Grunewaldt S. Riediger N. Dietrich C. (2007). Suitability of GFP-transformed isolates of the fungal root endophyte *Acremonium strictum* W. Gams for studies on induced Fusarium-wilt resistance in flax. Plant Root 1, 46–56. 10.3117/plantroot.1.46

[B29] Hernández-Ortiz P. Espeso E. A. (2013). Phospho-regulation and nucleocytoplasmic trafficking of CrzA in response to calcium and alkaline-pH stress in *Aspergillus nidulans*. Mol. Microbiol. 89, 532–551. 10.1111/mmi.12294 23772954

[B30] Hervás-Aguilar A. Penalva M. A. (2010). Endocytic machinery protein SlaB is dispensable for polarity establishment but necessary for polarity maintenance in hyphal tip cells of *Aspergillus nidulans*. Eukaryotic Cell 9, 1504–1518. 10.1128/EC.00119-10 20693304 PMC2950435

[B31] Hoagland D. R. Arnon D. I. (1950). The Water-Culture Method for Growing Plants Without Soil. Arnon, D. I. California Agricultural Experimental Station C347, 1-82. 1950. Berkeley, CA: College of Agriculture, University of California.

[B32] Kolar M. Punt P. J. van den Hondel C. A. Schwab H. (1988). Transformation of *Penicillium chrysogenum* using dominant selection markers and expression of an *Escherichia coli lacZ* fusion gene. Gene 62, 127–134. 10.1016/0378-1119(88)90586-0 3131191

[B33] Kosalková K. Marcos A. T. Fierro F. Hernando-Rico V. Gutiérrez S. Martín J. F. (2000). A novel heptameric sequence (TTAGTAA) is the binding site for a protein required for high level expression of pcbab, the first gene of the penicillin biosynthesis in *Penicillium chrysogenum*. J. Biol. Chem. 275, 2423–2430. 10.1074/jbc.275.4.2423 10644695

[B34] Larena I. De Cal A. Melgarejo P. (2007). Effects of stabilizers on shelf-life of *Epicoccum nigrum* formulations and their relationship with biocontrol of postharvest brown rot by *Monilinia* of peaches. J. Appl. Microbiol. 102, 570–582. 10.1111/j.1365-2672.2006.03075.x 17241364

[B35] Larena I. Melgarejo P. (2009). Development of a method for detection of the biocontrol agent *Penicillium oxalicum* strain 212 by combining PCR and a selective medium. Plant Dis. 93, 919–928. 10.1094/PDIS-93-9-0919 30754529

[B36] Larena I. Melgarejo P. De Cal A. (2002). Production, survival, and evaluation of solid-substrate inocula of *Penicillium oxalicum*, a biocontrol agent against fusarium wilt of tomato. Phytopathology 92, 863–869. 10.1094/PHYTO.2002.92.8.863 18942965

[B37] Larena I. Sabuquillo P. Melgarejo P. De Cal A. (2003). Biocontrol of Fusarium and Verticillium Wilt of tomato by *Penicillium oxalicum* under greenhouse and field conditions. J. Phytopathol. 151, 507–512. 10.1046/j.1439-0434.2003.00762.x

[B38] Lo C. T. Nelson E. B. Hayes C. K. Harman G. E. (1998). Ecological Studies of Transformed *Trichoderma harzianum* strain 1295–22 in the rhizosphere and on the phylloplane of creeping bentgrass. Phytopathology 88, 129–136. 10.1094/PHYTO.1998.88.2.129 18944981

[B39] Lübeck M. Knudsen I. M. B. Jensen B. Thrane U. Janvier C. Jensen D. F. (2002). GUS and GFP transformation of the biocontrol strain *Clonostachys rosea* IK726 and the use of these marker genes in ecological studies. Mycol. Res. 106, 815–826. 10.1017/S095375620200607X

[B40] Lu Z. Tombolini R. Woo S. Zeilinger S. Lorito M. Jansson J. K. (2004). *In vivo* study of *Trichoderma*-pathogen-plant interactions, using constitutive and inducible green fluorescent protein reporter systems. Appl. Environ. Microbiol. 70, 3073–3081. 10.1128/AEM.70.5.3073-3081.2004 15128569 PMC404383

[B41] Martinez-Beringola M. L. Salto T. Vázquez G. Larena I. Melgarejo P. De Cal A. (2013). *Penicillium oxalicum* reduces the number of cysts and juveniles of potato cyst nematodes. J. Appl. Microbiol. 115, 199–206. 10.1111/jam.12213 23560806

[B42] Melgarejo P. De Cal A. Larena I. Sabuquillo P. Guijarro B. (2005). Capítulo 3: agentes de control biológico de enfermedades. estrategias para el control biológico de hongos fitopatógenos,” in El Control Biológico de Plagas y Enfermedades. La Sostenibilidad de la Agricultura Mediterránea, eds Jacas J. Caballero P. Avilla J. (Castelló de la Plana: Univerdad Jaume I. Servicio de comunicación y publicaciones), 115–130.

[B43] Mikkelsen L. Sarrocco S. Lübeck M. Jensen D. F. (2003). Expression of the red fluorescent protein DsRed-Express in filamentous ascomycete fungi. FEMS Microbiol. Lett. 223, 135–139. 10.1016/S0378-1097(03)00355-0 12799012

[B44] Nahalkova J. Fatehi J. Olivain C. Alabouvette C. (2008). Tomato root colonization by fluorescent-tagged pathogenic and protective strains of *Fusarium oxysporum* in hydroponic culture differs from root colonization in soil. FEMS Microbiol. Lett. 286, 152–157. 10.1111/j.1574-6968.2008.01241.x 18657114

[B45] Nayak T. Szewczyk E. Oakley C. E. Osmani A. Ukil L. Murray S. L.. (2006). A versatile and efficient gene-targeting system for *Aspergillus nidulans*. Genetics 172, 1557. 10.1534/genetics.105.052563 16387870 PMC1456264

[B46] Osherov N. Mathew J. May G. S. (2000). Polarity-defective Mutants of *Aspergillus nidulans*. Fungal Genet. Biol. 31, 181–188. 10.1006/fgbi.2000.1236 11273680

[B47] Ossanna N. Mischke S. (1990). Genetic transformation of the biocontrol fungus *Gliocladium virens* to benomyl resistance. Appl. Environ. Microbiol. 56, 3052–3056. 16348312 10.1128/aem.56.10.3052-3056.1990 PMC184898

[B48] Pantazopoulou A. Peñalva M. A. (2009). Organization and dynamics of the Aspergillus nidulans Golgi during apical extension and mitosis. Mol. Biol. Cell 20, 4335–4347. 10.1091/mbc.e09-03-0254 19692566 PMC2762137

[B49] Pascual S. Rico J. R. De Cal A. Melgarejo P. (1997). Ecophysiological factors affecting growth, sporulation and survival of the biocontrol agent *Penicillium oxalicum*. Mycopathologia 139, 43–50. 10.1023/A:1006898724724 16283450

[B50] Peñalva M. A. (2005). Tracing the endocytic pathway of *Aspergillus nidulans* with FM4-64. Fungal Genet. Biol. 42, 963–975. 10.1016/j.fgb.2005.09.004 16291501

[B51] Peng G. Sutton J. C. (1991). Evaluation of microorganisms for biocontrol of *Botrytis cinerea* in strawberry. Can. J. Plant Pathol. 13, 247–257. 10.1080/07060669109500938

[B52] Picknett T. Saunders G. (1989). Transformation of *Penicillium chysogenum* with selection for increased resistance to benomyl. FEMS Microbiol. Lett. 60, 165–168. 10.1111/j.1574-6968.1989.tb03438.x 2506108

[B53] Skory C. D. Horng J. S. Pestka J. J. Linz J. E. (1990). Transformation of *Aspergillus parasiticus* with a homologous gene (*pyrG*) involved in pyrimidine biosynthesis. Appl. Environ. Microbiol. 56, 3315–3320. 2176447 10.1128/aem.56.11.3315-3320.1990 PMC184948

[B54] Snedecor G. Cochran W. (1980). Statistical Methods. Ames, IA: Iowa State University Press.

[B55] Suelmann R. Sievers N. Fischer R. (1997). Nuclear traffic in fungal hyphae: *in vivo* study of nuclear migration and positioning in *Aspergillus nidulans*. Mol. Microbiol. 25, 757–769. 10.1046/j.1365-2958.1997.5131873.x 9379904

[B56] Szewczyk E. Nayak T. Oakley C. E. Edgerton H. Xiong Y. Taheri-Talesh N.. (2006). Fusion PCR and gene targeting in *Aspergillus nidulans*. Nat. Protoc. 1, 3111–3120. 10.1038/nprot.2006.405 17406574

[B57] Thrane C. Lübeck M. Green H. Degefu Y. Allerup S. Jensen D. F. (1995). A tool for monitoring *Trichoderma harzianum*. I. Transformation with the GUS gene by protoplast technology. Phytopathology 85, 1428–1435. 10.1094/Phyto-85-1428

[B58] Tilburn J. Scazzocchio C. Taylor G. G. Zabicky-Zissman J. H. Lockington R. A. Davies R. W. (1983). Transformation by integration in *Aspergillus nidulans*. Gene 26, 205–221. 10.1016/0378-1119(83)90191-9 6368319

[B59] Ukil L. De Souza C. P. Liu H. L. Osmani S. A. (2009). Nucleolar separation from chromosomes during *Aspergillus nidulans* mitosis can occur without spindle forces. Mol. Biol. Cell 20, 2132–2145. 10.1091/mbc.e08-10-1046 19211837 PMC2669022

[B60] van Hartingsveldt W. Mattern I. van Zeijl C. Pouwels P. van den Hondel C. (1987). Development of a homologous transformation system for *Aspergillus niger* based on the *pyrG* gene. Mol. Gen. Genet. 206, 71–75. 10.1007/BF00326538 3472035

[B61] Villarino M. De C. A. Melgarejo P. Larena I. Espeso E. A. (2016). The development of genetic and molecular markers to register and commercialize *Penicillium rubens* (formerly *Penicillium oxalicum*) strain 212 as a biocontrol agent. Microb. Biotechnol. 9, 89–99. 10.1111/1751-7915.12325 26467970 PMC4720407

[B62] Whitehead M. Unkles S. Ramsden M. Campbell E. Gurr S. Spence D. (1989). Transformation of a nitrate reductase deficient mutant of *Penicillium chrysogenum* with the corresponding *Aspergillus niger* and *a*. nidulans niaD genes. Mol. Gen. Genet. 216, 408–411. 10.1007/BF00334383

[B63] Woloshuk C. P. Seip E. R. Payne G. A. Adkins C. R. (1989). Genetic transformation system for the aflatoxin-producing fungus *Aspergillus flavus. Appl. Environ*. Microbiol. 55, 86–90. 10.1128/aem.55.1.86-90.1989 PMC184058 2495764

[B64] Yang L. Ukil L. Osmani A. Nahm F. Davies J. De Souza C. P. C.. (2004). Rapid production of gene replacement constructs and generation of a green fluorescent protein-tagged centromeric marker in *Aspergillus nidulans*. Eukaryotic Cell 3, 1359–1362. 10.1128/EC.3.5.1359-1362.2004 15470263 PMC522605

[B65] Zheng Y. M. Lin F. L. Gao H. Zou G. Zhang J. W. Wang G. Q.. (2017). Development of a versatile and conventional technique for gene disruption in filamentous fungi based on CRISPR-Cas9 technology. Sci. Rep. 7, 9250. 10.1038/s41598-017-10052-3 28835711 PMC5569088

