# Supplementary material for: Transformation of Penicillium rubens 212 and Expression of GFP and DsRED Coding Genes for Visualization of Plant-Biocontrol Agent Interaction
Source: Front Microbiol. 2018 Jul 23;9:1653. doi: 10.3389/fmicb.2018.01653 (PMC6064719; doi:10.3389/fmicb.2018.01653)
Supplement: Figure S1 — Schematic representation of plasmids used in this work. Autoreplicative plasmids have a pUC19 backbone, and integrative plasmids are of TOPO series (pCRII-TOPO). AMA1 indicate presence of autonomous replicative sequences. As selection markers for P. rubens transformation, plasmids carry either pyr-4 or pyrG genes from N. crassa and A. fumigatus, respectively. Indicated are the presence of chimaeric transgenes: GA5xGFP, without promoter or gpdAp (glyceraldehyde 3-P dehydrogenase promoter). DsRed was expressed under gpdAp and also carries the terminator from trpC gene, trpCter. [file Image_1.PDF]

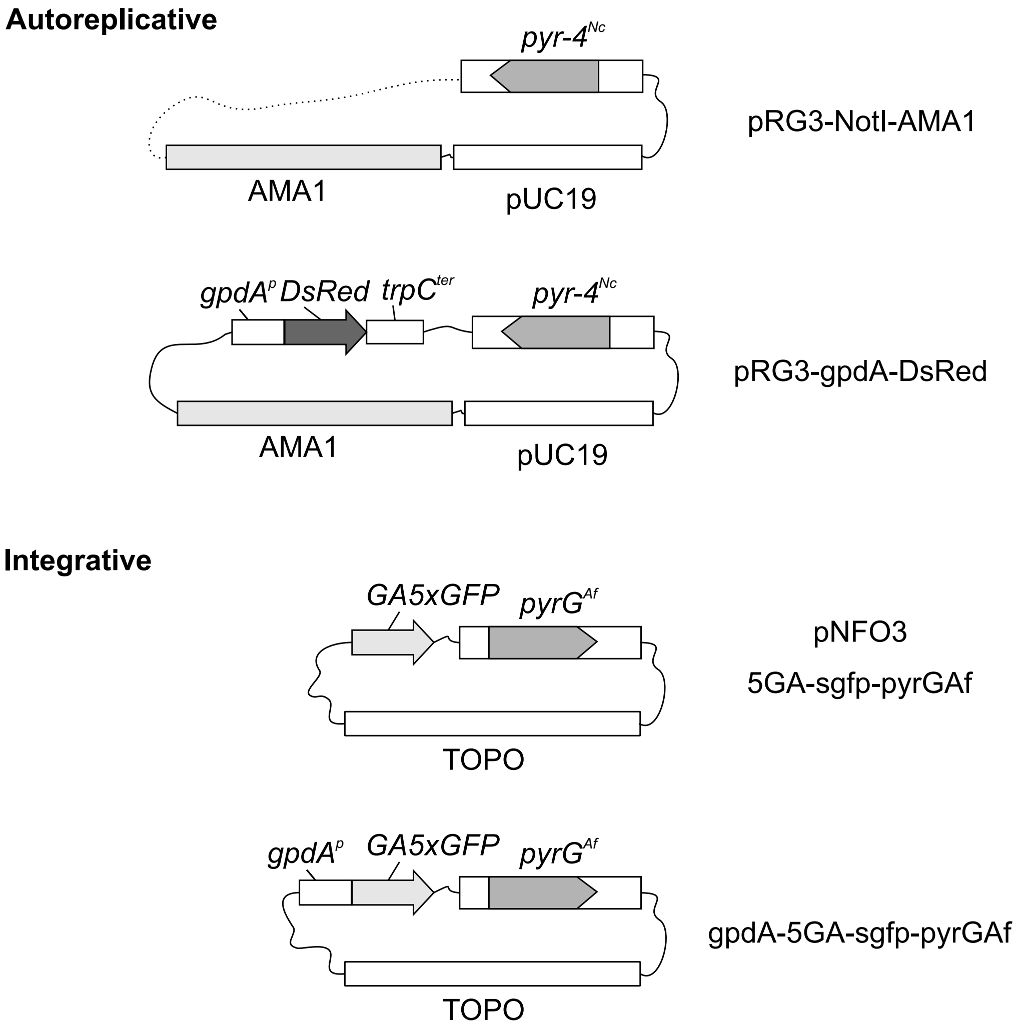

FIG S1 Schematic representation of plasmids used in this work. Autoreplicative plasmids have a pUC19 backbone, and integrative plasmids are of TOPO series (pCRII-TOPO). AMA1 indicate presence of autonomous replicative sequences. As selection markers for *P. rubens* transformation, plasmids carry either *pyr-4* or *pyrG* genes from *N. crassa* and *A. fumigatus*, respectively. Indicated are the presence of chimaeric transgenes: GA5xGFP, without promoter or $gpdA^{p}$ (glyceraldehyde 3-P dehydrogenase promoter). DsRed was expressed under $gpdA^{p}$ and also carries the terminator from *trpC* gene, $trpC^{ter}$.
