# Supplementary material for: Transformation of Penicillium rubens 212 and Expression of GFP and DsRED Coding Genes for Visualization of Plant-Biocontrol Agent Interaction
Source: Front Microbiol. 2018 Jul 23;9:1653. doi: 10.3389/fmicb.2018.01653 (PMC6064719; doi:10.3389/fmicb.2018.01653)
Supplement: Figure S3 — Effect of temperature and pH on the radial growth of wild-type PO212 and the transformed PO212 strain PO212_inGFP9. Diameter of colonies grown at the indicated temperatures and pH values on PDA are represented along 14 days of incubation. Values are the means of 10 replications. Standard errors are shown. Strains code: PO212, PO212_inGFP9. [file Image_3.PDF]

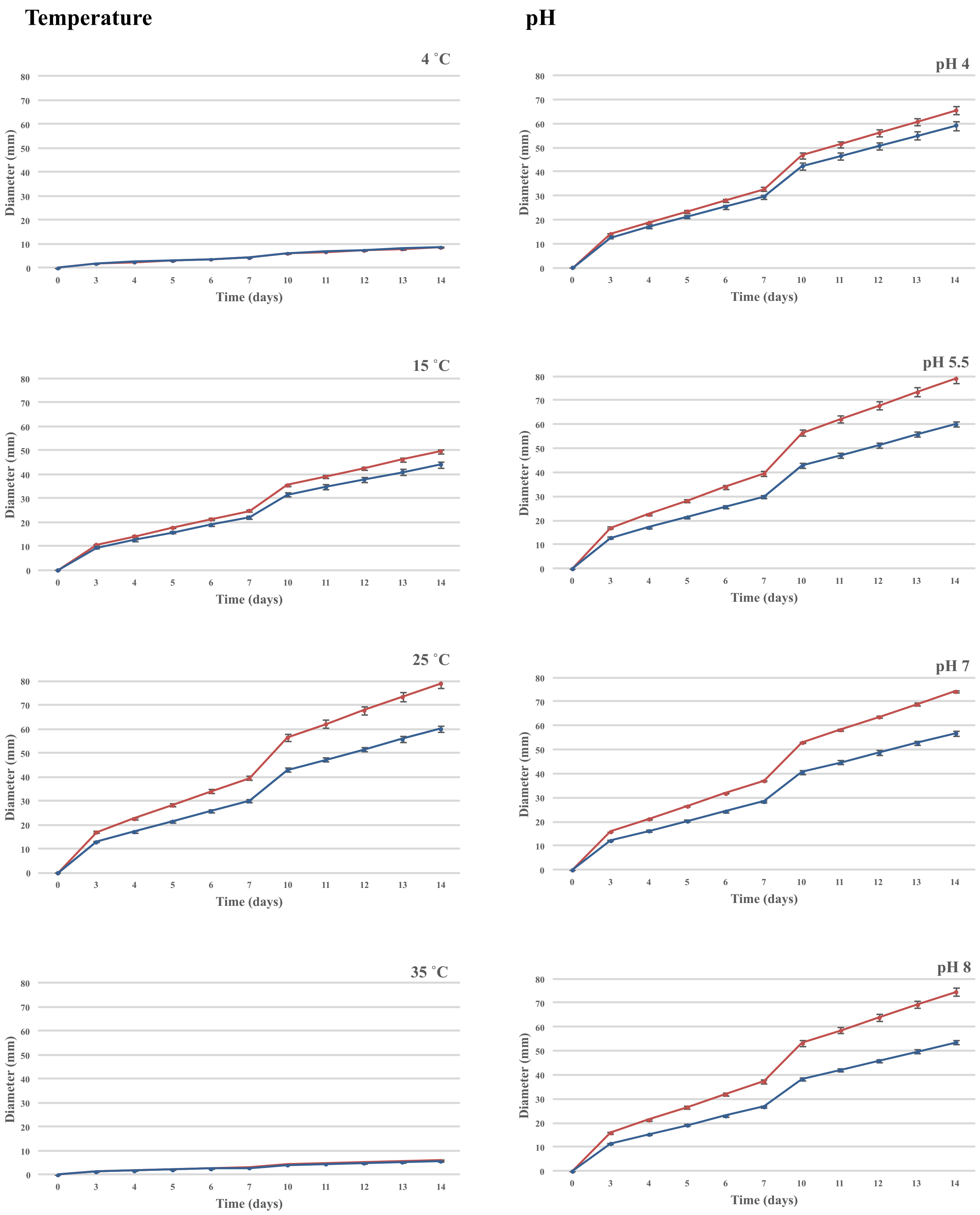

FIG S3 Effect of temperature and pH on the radial growth of wild-type PO212 and the transformed PO212 strain PO212_inGFP9. Diameter of colonies grown at the indicated temperatures and pH values on PDA are represented along 14 days of incubation. Values are the means of 10 replications. Standard errors are shown. Strains code: PO212 (red line), PO212_inGFP9 (blue line)
