# Supplementary material for: Transformation of Penicillium rubens 212 and Expression of GFP and DsRED Coding Genes for Visualization of Plant-Biocontrol Agent Interaction
Source: Front Microbiol. 2018 Jul 23;9:1653. doi: 10.3389/fmicb.2018.01653 (PMC6064719; doi:10.3389/fmicb.2018.01653)
Supplement: Table S1 — Comparison of the germination percentage (%) of the wild-type PO212 (wtPO212) and the transformed PO212 strains (PO212_ar1, PO212_arRED3, and PO212_in5) at different temperature (25 and 35°C) and pH (4, 5.5, 7, and 8). [file Table_1.docx]

**TABLE S1** Comparison of the germination percentage (%) of the wild-type PO212 (wtPO212) and the transformed PO212 strains (PO212_ar1, PO212_arRED3 and PO212_in5) at different temperatures and pH values

| Strain | Temperature (ºC) | |  | pH | | | |
| --- | --- | --- | --- | --- | --- | --- | --- |
|  | 25 | 35 |  | 4 | 5.5 | 7 | 8 |
| wtPO212 | 73.8 | 0.0 |  | 81.3 a | 84.7 | 73.8 | 82.2 |
| PO212_ar1 | 63.8 | 0.0 |  | 71.6 ab | 74.0 | 63.8 | 76.2 |
| PO212_arRED3 | 67.6 | 17.6 |  | 64.0 b | 72.4 | 67.6 | 70.4 |
| PO212_in5 | 72.0 | 35.1 |  | 84.2 a | 83.6 | 72.0 | 71.1 |
| MS_within_ | 183.3  NS | 362.9  NS |  | 42.4 | 31.4  NS | 183.3  NS | 24.5  NS |

Data are displayed as the mean of three replications. Means followed by the same letter in each column are not significantly different by Student Newman Keul´s test (P≤0.05). The repeat confirmed the results, so only results from one repeat are shown. MS_within_ — error mean square. NS—not significant.
