# Supplementary material for: Transformation of Penicillium rubens 212 and Expression of GFP and DsRED Coding Genes for Visualization of Plant-Biocontrol Agent Interaction
Source: Front Microbiol. 2018 Jul 23;9:1653. doi: 10.3389/fmicb.2018.01653 (PMC6064719; doi:10.3389/fmicb.2018.01653)
Supplement: Table S2 — Comparison of the germination percentage (%) of the wild-type PO212 (wtPO212) and the transformed PO212 strain, PO212_inGFP9 at different temperatures and pH values. [file Table_2.PDF]

TABLE S2 Comparison of the germination percentage (%) of the wild-type PO212 (wtPO212) and the transformed PO212 strain, PO212_inGFP9 at different temperatures and pH values

Strain	Temperature (ºC)			pH			
	15	25	35	4	5.5	7	8
wtPO212	0.4 (0.2)	77.9	0.2	88.1	86.6	74.1	13.8 b
PO212_inGFP9	10.9 (1.5)	76.3	0.0	78.5 a	82.9	60.5	27.4 a
MS_{within}	(1.1)	35.7	0.1	135.9	157.0	241.2	107.4
	NS	NS	NS	NS	NS	NS	

Data are displayed as the mean of three replications. Means followed by the same letter in each column are not significantly different by Student Newman Keul´s test (P≤0.05). The repeat for temperature confirmed the results so, only results from one repeat are shown. Each value for pH is the average of two assays, with three replications per assay. Data in parentheses were subjected to asin (x/100) transformation. MS_{within} — error mean square. NS—not significant.
