# Supplementary material for: Transformation of Penicillium rubens 212 and Expression of GFP and DsRED Coding Genes for Visualization of Plant-Biocontrol Agent Interaction
Source: Front Microbiol. 2018 Jul 23;9:1653. doi: 10.3389/fmicb.2018.01653 (PMC6064719; doi:10.3389/fmicb.2018.01653)
Supplement: Table S3 — Comparison of the length of the germ tube (μm) of the wild-type PO212 (wtPO212) and the transformed PO212 strains (PO212_ar1, PO212_arRED3, and PO212_in5) at different temperatures and pH values. [file Table_3.PDF]

TABLE S3 Comparison of the length of the germ tube (µm) of the wild-type PO212 (wtPO212) and the transformed PO212 strains (PO212_ar1, PO212_arRED3 and PO212_in5) at different temperatures and pH values

Strain	Temperature (ºC)		pH			
	25	35	4	5.5	7	8
wtPO212	26.6	0.0	36.5 ab	48.6	26.6	36.5 a
PO212_ar1	22.5	0.0	30.0 b	38.7	22.5	35.0 a
PO212_arRED3	26.3	4.3	33.3 ab	35.0	26.3	29.9 b
PO212_in5	27.7	7.4	39.4 a	33.8	27.7	22.4 c
MS_{within}	30.1	22.1	12.1	40.7	30.1	4.3
	NS	NS		NS	NS	

Data are displayed as the mean of three replications. Data were analyzed by analysis of variance using the general linear model procedure in Statgraphics XVI Centurion for Windows 7 (StatPoint, Inc. Herndon, VA, USA). The repeat for temperature confirmed the results so, only results from one repeat are shown. Each value for pH is the average of two assays, with three replications per assay. Means followed by the same letter in each column are not significantly different by Student Newman Keul´s test (P≤0.05). MS_{within} — error mean square. NS—not significant.
