# Supplementary material for: Transformation of Penicillium rubens 212 and Expression of GFP and DsRED Coding Genes for Visualization of Plant-Biocontrol Agent Interaction
Source: Front Microbiol. 2018 Jul 23;9:1653. doi: 10.3389/fmicb.2018.01653 (PMC6064719; doi:10.3389/fmicb.2018.01653)
Supplement: Table S4 — Comparison of the length of the germ tube (μm) of the wild-type PO212 (wtPO212) and the transformed PO212 strain, PO212_inGFP9 at different temperatures and pH values. [file Table_4.docx]

**TABLE S4** Comparison of the length of the germ tube (µm) of the wild-type PO212 (wtPO212) and the transformed PO212 strain, PO212_inGFP9 at different temperatures and pH values

| Strain | Temperature (ºC) | | |  | pH | | | |
| --- | --- | --- | --- | --- | --- | --- | --- | --- |
|  | 15 | 25 | 35 |  | 4 | 5.5 | 7 | 8 |
| PO212 | 3.8 | 20.9 | 1.1 |  | 18.5 | 34.1 | 20.9 | 15.5 |
| PO212_inGFP9 | 3.3 | 24.1 | 0.0 |  | 18 | 28.1 | 24.1 | 17.3 |
| MS_within_ | 24.7  NS | 39.7  NS | 1.7  NS |  | 5.2  NS | 45.3  NS | 39.7  NS | 11.9  NS |

Data are displayed as the mean of three replications. Each value is the average of two assays, with three replications per assay. MS_within_ — error mean square. NS—not significant.
